# The Influence of Pre-Existing Beta-Blockers Use on Survival Outcomes in HER2 Positive Advanced Breast Cancer: Pooled Analysis of Clinical Trial Data

**DOI:** 10.3389/fonc.2020.01130

**Published:** 2020-07-14

**Authors:** Natansh D. Modi, Jin Quan Eugene Tan, Andrew Rowland, Bogda Koczwara, Ganessan Kichenadasse, Ross A. McKinnon, Michael D. Wiese, Michael J. Sorich, Ashley M. Hopkins

**Affiliations:** ^1^College of Medicine and Public Health, Flinders University, Adelaide, SA, Australia; ^2^Department of Medical Oncology, Flinders Medical Centre, Adelaide, SA, Australia; ^3^School of Pharmacy and Medical Sciences, University of South Australia, Adelaide, SA, Australia

**Keywords:** beta-blockers, survival, breast cancer, HER2 positive, targeted therapy

## Abstract

**Introduction:** Beta-blockers (BB) are commonly used to manage cardiovascular disease and may have benefits in controlling complications of anti-HER2 therapies. This study aimed to evaluate the association of pre-existing BB use with survival outcomes in patients initiating anti-HER2 therapy for advanced breast cancer (ABC).

**Materials and Methods:** Data from clinical trials EMILIA, TH3RESA, MARIANNE, and CLEOPATRA was pooled. Cox proportional analysis was used to assess the association between pre-existing BB use with survival outcomes in patients initiating anti-HER2 therapies.

**Results:** Of the 2,777 patients with HER2 positive ABC, 266 were using a BB at the time of anti-HER2 therapy initiation. BB use was associated with worse overall survival (OS) (adjusted HR = 1.27, 95% CI: 1.04–1.55). Sensitivity analysis in patients with pre-existing cardiovascular disease (CVD) also indicated that BB use was associated with worse OS (1.29, 1.02–1.63).

**Conclusion:** In large high-quality data, BB use at the time of anti-HER2 therapy initiation for ABC was independently associated with worse OS, regardless of CVD status. The finding is contrary to pre-study hypotheses and findings in other BC subtypes. Future research should aim to gain a deeper understanding of the effects of BBs on specific BC subtypes, cancer types, and cancer treatments.

## Introduction

Human epidermal growth factor receptor 2 (HER2) amplification is associated with an increased incidence of metastasis in breast cancer (BC) (1). Monoclonal antibodies targeting HER2 and associated pathways, such as trastuzumab, have revolutionized the standard of care for patients with both early and advanced HER2 positive BC (2, 3). Whilst, anti-HER2 therapies are generally well-tolerated, they are associated with cardiotoxic effects, which may be further augmented when used in combination with anthracyclines (4, 5).

Pre-clinical evidence indicates that beta-blockers (BB) may have anticancer and immune-boosting effects, and clinical trials investigating the effects of BB on cancer are being initiated (6, 7). Guidelines also support the prophylactic use of BB to reduce anti-HER2 therapy-induced cardiotoxicities (8, 9). Specifically, BB may suppress the effects of catecholamine released through the stress associated activation of the sympathetic nervous system, which has been linked to cell survival, proliferation, and motility (10). Additionally, stimulation of β2 receptors may promote resistance to anti-HER2 therapies; thus it has also been hypothesized that BB may re-sensitize patients to anti-HER2 therapy (11). Despite this, the effects of BB have not been investigated in patients with HER2 positive advanced breast cancer (ABC) within large high-quality data. This is important as research has indicated that BB effects may not be the same between cancer subtypes and treatments (12–14).

This study aimed to evaluate the association of pre-existing oral BB use with overall survival (OS) and progression-free survival (PFS) in patients with HER2 positive ABC initiating anti-HER2 therapies.

## Materials and Methods

### Patient Population

Individual participant data (IPD) from the Roche sponsored phase III clinical trials CLEOPATRA (NCT00567190) (15–17), MARIANNE (NCT01120184) (18, 19), EMILIA (NCT00829166) (20, 21) and TH3RESA (NCT01419197) (22, 23) was utilized in this retrospective pooled analysis study.

All 4 trials were randomized controlled trials (RCT) and included patients with locally recurrent, unresectable or metastatic HER2 positive BC (15–23). CLEOPATRA and MARIANNE included treatment naïve patients (not accounting hormonal patients) (15–19). CLEOPATRA patients were randomly assigned in a 1:1 ratio to receive either placebo or pertuzumab, with trastuzumab plus docetaxel (15–17). Whilst, MARIANNE participants were randomly assigned 1:1:1 to trastuzumab plus a taxane, trastuzumab emtansine (T-DM1) plus placebo, or T-DM1 plus pertuzumab (18, 19). EMILIA and TH3RESA included patients with documented progression of disease despite prior therapies (20–23). Participants were randomly assigned 1:1 to either lapatinib plus capecitabine, or T-DM1 in EMILIA (20, 21). While in TH3RESA patients were randomly assigned 1:2 to the treatment of physician's choice, or T-DM1(22, 23).

The inclusion criteria for each of the trials included left ventricular ejection fraction (LVEF) ≥ 50%.

Participants of EMILIA and TH3RESA who respectively received either lapatinib plus capecitabine or treatment of physician's choice were excluded from the current pooled analyses as the study aimed to evaluate the effect of concomitant oral BB use on survival outcomes in patients initiating trastuzumab backboned therapies (i.e., trastuzumab, pertuzumab, and T-DM1 are associated with cardiotoxic effects).

### Predictors and Outcomes

The primary assessed outcome was OS, with PFS assessed as a secondary outcome. OS was defined as the time from randomization to the last follow-up visit or death from any cause in all studies. PFS was defined as the time from randomization to disease progression or death from any cause, with progression assessed by the investigators using the Response Evaluation Criteria in Solid Tumors (RECIST) version 1.0 within CLEOPATRA and EMILIA while RECIST version 1.1 within MARIANNE and TH3RESA.

Across the four pooled clinical trials, available pre-treatment characteristic data included BB use status, age, body mass index (BMI; Normal, Overweight, Obese or Underweight), race (Asian or Non-Asian), presence of brain metastasis and visceral disease, albumin below the lower limit of normal (< LLN), Eastern Cooperative Oncology Group performance status (ECOG PS), estrogen/progesterone receptor status (ER/PR), any prior taxane, anthracycline or trastuzumab use, presence of arrhythmia, heart failure, cerebrovascular disease, hypertension, coronary artery disease, other cardiovascular diseases (CVD) or diabetes mellitus—this allowed examination of BB use in the univariable and adjusted analysis.

Secondary analysis of anonymized shared clinical trial data was confirmed negligible risk research by the Southern Adelaide Local Health Network, Office for Research and Ethics, and was exempted from review.

### Statistical Analysis

Cox proportional hazard analysis was used to assess the association between pre-existing concomitant BB use with OS and PFS. Results were reported as hazard ratios (HR) with 95% confidence intervals (95%CI). Statistical significance was set at a threshold of *P* < 0.05 and was determined via the likelihood ratio test. All analyses were stratified by study and treatment. Regression adjusted analyses by age, BMI, race, presence of brain metastasis and visceral disease, albumin, ECOG PS, ER/PR status, any prior taxane, anthracycline or trastuzumab use, presence of arrhythmia, heart failure, cerebrovascular disease, hypertension, coronary artery disease, other CVD or diabetes mellitus were conducted. Analyses using doubly robust estimation—[regression model adjustment plus propensity score weighting adjustment (propensity score estimated using logistic regression)] were undertaken to confirm identified associations (24).

Sensitivity analysis of the association between pre-existing BB use with OS and PFS in patients with pre-existing CVD at baseline was conducted.

Kaplan-Meier analysis was used for plotting and estimating OS/PFS probabilities. All analyses were conducted using R version 3.4.3.

## Results

### Patient Population

Data was available from 2,777 patients (Supplementary Table 1), of which 266 (10%) were using an oral BB at the time of anti-HER2 therapy initiation. Supplementary Table 2 presents patient characteristic data by BB use status. Median follow-up was 50 [95% CI: 49–51] months in CLEOPATRA, 35 [34–36] months in MARIANNE, 47 [46–49] months in EMILIA and 35 [34–36] months in TH3RESA. Within the total 2,777 patients, 762 had pre-existing CVD at the time of anti-HER2 therapy initiation, of which 217 (29%) were using a BB.

Of the total 266 patients using a BB, 212 were using a selective BB, 51 a non-selective BB, and three were using a BB with intrinsic sympathomimetic activity (ISA). Bisoprolol (*n* = 68), atenolol (*n* = 67), metoprolol (*n* = 57), carvedilol (*n* = 23), and propranolol (*n* = 21) were the most used BB. Of the total 266 (10%) patients using a BB, 60 (7%) were in CLEOPATRA, 103 (10%) in MARIANNE, 51 (10%) in EMILIA and 52 (13%) in TH3RESA (P[χ^2^] = 0.021).

### Association Between Concomitant BB Use and Survival

BB use was associated with worse OS (adjusted HR= 1.27, 95% CI:1.04–1.55). No statistically significant association between BB use with PFS was identified (adjusted HR = 1.10, 95% CI: 0.92–1.30) (Table 1). On univariable analysis, similar associations between BB use with OS and PFS were observed (Supplementary Table 3). Further, doubly robust estimation produced similar associations between BB use with OS (HR= 1.35, 95% CI: 1.03–1.77) and PFS (HR= 1.18, 95% CI: 0.94–1.49).

**Table 1:** Adjusted analysis of pre-existing BB use with OS and PFS in the pooled cohort.

**CVD Cohort**		**Overall Survival**		**Progression-Free Survival**	
	***N***	**HR [95% CI]**	***P***	**HR [95% CI]**	***P***
Beta-blocker use^1^			0.022		0.291
No	2,511	1		1	
Yes	266	1.27 [1.04–1.55]		1.10 [0.92–1.30]	

^1^*Adjustment Variables: Age, Race, BMI, Albumin count, ECOG PS, ER/PR Status, Presence of Visceral Disease and Brain Metastasis, Arrhythmia, Coronary Artery Disease, Heart Failure, Hypertension, Cerebrovascular disease, Diabetes and Other Cardiovascular diseases, Prior treatment to anthracyclines, taxanes and trastuzumab*.

*CI, Confidence interval; HR, Hazard ratio; N, Number of subjects; ECOG PS, Eastern cooperative oncology group performance status; BMI, Body Mass Index; ER/PR, Estrogen Receptor/Progesterone Receptor*.

**Figure 1:**
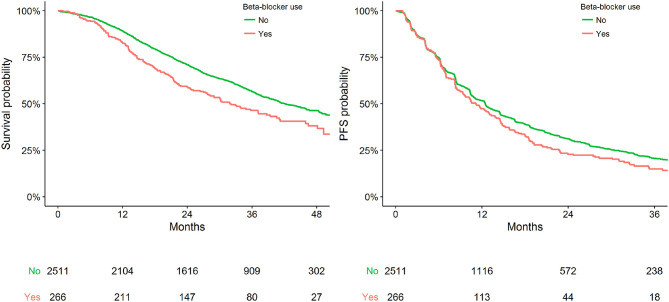
Kaplan-Meier plot representing survival outcomes in the pooled cohort by status of BB use.

Figure 1 presents Kaplan-Meier estimates of OS and PFS by the status of BB use. No substantial heterogeneity in effect size of BB use was apparent between studies or treatment (Supplementary Table 4).

### Sensitivity Analysis

**Figure 2:**
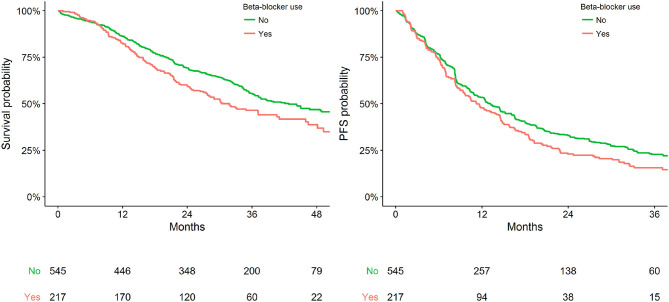
Kaplan-Meier plot representing survival outcomes in patients with pre-existing CVD by status of BB use.

Within the available population, 762 patients had pre-existing CVD at the time of anti-HER2 therapy initiation, of which 217 (29%) were using a BB. BB use was significantly associated with worse OS in the sensitivity cohort (adjusted HR = 1.29, 95% CI: 1.02–1.63). No statistically significant association between BB use with PFS was identified (adjusted HR = 1.04, 95% CI: 0.85–1.26) (Table 2). Figure 2 presents Kaplan Meier estimates of OS and PFS by the status of BB use in patients with pre-existing CVD.

**Table 2:** Adjusted analysis of pre-existing BB use with OS and PFS in the cardiovascular disease cohort.

**CVD Cohort**		**Overall Survival**		**Progression-Free Survival**	
	***N***	**HR [95% CI]**	***P***	**HR [95% CI]**	***P***
Beta-blocker use^1^			0.036		0.730
No	545	1		1	
Yes	217	1.29 [1.02–1.63]		1.04 [0.85–1.26]	

^1^*Adjustment Variables: Age, Race, BMI, Albumin count, ECOG PS, ER/PR Status, Presence of Visceral Disease and Brain Metastasis, Coronary Artery Disease, Cerebrovascular disease, Arrhythmia, Hypertension, other cardiovascular diseases and Diabetes comorbidity, Prior treatment to anthracyclines, taxanes and trastuzumab*.

*CI, Confidence interval; HR, Hazard ratio; N, Number of subjects; ECOG PS, Eastern cooperative oncology group performance status; BMI, Body Mass Index; ER/PR, Estrogen Receptor/Progesterone Receptor*.

## Discussion

In *post-hoc* analysis of large high-quality data from prospective clinical trials, pre-existing oral BB use was independently associated with inferior OS in ABC patients initiating anti-HER2 treatments. Inferior OS was maintained in analyses adjusted for CVD and sensitivity subgroup analysis of those with pre-existing CVD.

Prior studies have investigated the effect of pre-existing BB use on survival outcomes in patients initiating treatments for early BC and advanced triple negative-breast cancer (TNBC). However, little is known about BB effects in patients with HER2 positive ABC. In advanced TNBC, BB use has demonstrated associations with improved survival outcomes in retrospective analyses of clinical trial and patient medical data (10, 25). Further, contemporary evidence supports the use of BB for protection from cardiotoxic regimens (8, 26). Specifically, an RCT indicated that post-diagnostic BB use was associated with improved cardiotoxic free survival compared to placebo in patients initiating trastuzumab and anthracycline therapy for HER 2 positive non-metastatic BC (26).

Despite prior findings in other BC subtypes, in our study of patients initiating anti-HER2 therapy for HER2 positive ABC, the pre-existing use of a BB was independently associated with worse OS. The biological basis of these findings remains unknown, despite adjusting analyses for age, BMI, race, presence of brain metastasis and visceral disease, albumin, ECOG PS, ER/PR status, any prior taxanes, anthracycline or trastuzumab use, and the presence of hypertension, heart failure, coronary artery disease, cerebrovascular disease, arrhythmia, other CVDs or diabetes mellitus. Furthermore, the association was observed in a sensitivity analysis of patients with pre-existing CVD. Whilst the analyses have been adjusted there may be still confounders, for example, the frailty of non-BB users.

Another future research interest is possible differences in the effects of non-selective vs. selective BB on cancer care. Biological studies suggest that antagonism of β2 receptors is responsible for the anti-cancer effects of BB rather than β1 antagonism. Hence non-selective BB could be more effective in improving survival outcomes (11). Prolonged BB exposure can also lead to β receptor upregulation, hampering the effectiveness of BB (10). This phenomenon has been demonstrated in observational studies where post-diagnostic BB use was associated with significant improvements in survival outcomes compared to pre-diagnostic BB use (27). In the present study, the sample of patients on non-selective BB was insufficient for an appropriately powered subgroup analysis (*n* = 51), and data on the length of prior BB use was not available.

A key limitation of this study is the unplanned *post-hoc* nature of the analysis (28). Nonetheless, this study pooled large (*n* = 2,777) and high-quality data from four contemporary RCTs (CLEOPATRA, MARIANNE, TH3RESA, and EMILIA), increasing study power and generalizability. Despite this, the sample was insufficient to allow subgroup analysis of BB effects by specific CVD types (e.g., arrhythmia, coronary artery disease). Furthermore, a lack of BB dose data inhibited exploratory analysis of the dose-response relationship was not possible (29, 30). While BB use was independently and significantly associated with worsened OS in the total and sensitivity cohort, the associations with PFS was not statistically significant. The reasons for this discrepancy are unclear, however, PFS is an imperfect surrogate that may be affected by variability in the timing of assessments, investigators, and measurement biases (31, 32). Additionally, OS associations may be confounded by subsequent therapies or cross over (31, 32). This indicates that the effect of BB may be compounded in patients with HER2 positive ABC rather than isolated to therapies including trastuzumab.

In large high-quality data, pre-existing use of BB in HER2 positive ABC patients initiating anti-HER2 therapies was independently associated with worse OS. The finding is contrary to pre-study hypotheses, findings in other BC subtypes, and emerging preclinical data for effects of BB on cancer. While this study does not justify the cessation of BB, and CVD should continue to be appropriately treated within HER2 positive ABC, the study presents important evidence that the effect of BB may not be the same between cancers or BC subtypes—with the impact potentially being negative within HER2 positive ABC. Future research is urgently required to validate findings in large real-world databases and prospective studies. Subsequent studies should aim to gain a deeper understanding of the effects of BB while considering dose responses, duration of prior exposure, and BB selectivity on specific BC subtypes, cancer types, and cancer treatments.

## Data Availability Statement

Publicly available datasets were analyzed in this study. This data can be found here: clinicalstudydatarequest.com.

## Ethics Statement

The study was an independent secondary analysis of anonymized shared clinical trial data. Secondary analysis of anonymized shared clinical trial data was confirmed negligible risk research by the Southern Adelaide Local Health Network, Office for Research and Ethics and was exempt from review. Data were accessed according to Roche's policy and process for clinical study data sharing. Additionally, these trials which were conducted by Roche were performed in accordance with Good Clinical Practice guidelines and the provisions of the Declaration of Helsinki. Protocol approval was obtained from an independent ethics committee at each study site. All patients provided written informed consent.

## Author Contributions

All authors contributed to the article and approved the submitted version.

## Conflict of Interest

AR, MS, and RM report grants from Pfizer, outside the submitted work. The remaining authors declare that the research was conducted in the absence of any commercial or financial relationships that could be construed as a potential conflict of interest.
