# Supplementary material for: The Influence of Pre-Existing Beta-Blockers Use on Survival Outcomes in HER2 Positive Advanced Breast Cancer: Pooled Analysis of Clinical Trial Data
Source: Front Oncol. 2020 Jul 14;10:1130. doi: 10.3389/fonc.2020.01130 (PMC7373122; doi:10.3389/fonc.2020.01130)
Supplement: Supplementary file 1 [file Data_Sheet_1.docx]

**Supplementary Tables:**

Supplementary Table 1: Demographic data

|  | Total | CLEOPATRA | EMILIA | MARIANNE | TH3RESA |
| --- | --- | --- | --- | --- | --- |
|  | No. 2,777 | No. 804 | No. 490 | No. 1,080 | No. 403 |
| The actual treatment given | | | | | |
| Pertuzumab + Trastuzumab + Docetaxel | 408 (15%) | 408 (51%) | 0 (0%) | 0 (0%) | 0 (0%) |
| Pertuzumab + Trastuzumab emtansine | 366 (13%) | 0 (0%) | 0 (0%) | 366 (34%) | 0 (0%) |
| Placebo + Trastuzumab + Docetaxel | 396 (14%) | 396 (49%) | 0 (0%) | 0 (0%) | 0 (0%) |
| Placebo + Trastuzumab emtansine | 361 (13%) | 0 (0%) | 0 (0%) | 361 (33%) | 0 (0%) |
| Trastuzumab + Docetaxel/Paclitaxel | 353 (13%) | 0 (0%) | 0 (0%) | 353 (33%) | 0 (0%) |
| Trastuzumab emtansine | 893 (32%) | 0 (0%) | 490 (100%) | 0 (0%) | 403 (100%) |
| Study | | | | | |
| CLEOPATRA | 804 (29%) | 804 (100%) | 0 (0%) | 0 (0%) | 0 (0%) |
| EMILIA | 490 (18%) | 0 (0%) | 490 (100%) | 0 (0%) | 0 (0%) |
| MARIANNE | 1,080 (39%) | 0 (0%) | 0 (0%) | 1,080 (100%) | 0 (0%) |
| TH3RESA | 403 (15%) | 0 (0%) | 0 (0%) | 0 (0%) | 403 (100%) |
| Age | | | | | |
| <=60 | 2,068 (74%) | 596 (74%) | 381 (78%) | 784 (73%) | 307 (76%) |
| >60 | 709 (26%) | 208 (26%) | 109 (22%) | 296 (27%) | 96 (24%) |
| Race | | | | | |
| Non-Asian | 2,113 (76%) | 542 (67%) | 398 (81%) | 827 (77%) | 346 (86%) |
| Asian | 663 (24%) | 261 (32%) | 92 (19%) | 253 (23%) | 57 (14%) |
| Missing | 1 (0%) | 1 (0%) | 0 (0%) | 0 (0%) | 0 (0%) |
| BMI - WHO classification | | | | | |
| Normal | 1,199 (43%) | 347 (43%) | 208 (42%) | 454 (42%) | 190 (47%) |
| Obese | 627 (23%) | 186 (23%) | 119 (24%) | 244 (23%) | 78 (19%) |
| Overweight | 846 (30%) | 241 (30%) | 147 (30%) | 346 (32%) | 112 (28%) |
| Underweight | 74 (3%) | 29 (4%) | 8 (2%) | 30 (3%) | 7 (2%) |
| Missing | 31 (1%) | 1 (0%) | 8 (2%) | 6 (1%) | 16 (4%) |
| ECOG PS | | | | | |
| 0 | 1,703 (61%) | 519 (65%) | 298 (61%) | 707 (65%) | 179 (44%) |
| >=1 | 1,071 (39%) | 285 (35%) | 191 (39%) | 373 (35%) | 222 (55%) |
| Missing | 3 (0%) | 0 (0%) | 1 (0%) | 0 (0%) | 2 (0%) |
| Brain metastasis | | | | | |
| Absent | 2,676 (96%) | 803 (100%) | 450 (92%) | 1,065 (99%) | 358 (89%) |
| Present | 98 (4%) | 1 (0%) | 40 (8%) | 13 (1%) | 44 (11%) |
| Missing | 3 (0%) | 0 (0%) | 0 (0%) | 2 (0%) | 1 (0%) |
| Albumin | | | | | |
| < LLN | 268 (10%) | 68 (8%) | 39 (8%) | 102 (9%) | 59 (15%) |
| >= LLN | 2,455 (88%) | 708 (88%) | 448 (91%) | 968 (90%) | 331 (82%) |
| Missing | 54 (2%) | 28 (3%) | 3 (1%) | 10 (1%) | 13 (3%) |
| Visceral Disease | | | | | |
| Absent | 778 (28%) | 176 (22%) | 161 (33%) | 339 (31%) | 102 (25%) |
| Present | 1,999 (72%) | 628 (78%) | 329 (67%) | 741 (69%) | 301 (75%) |
| ER & PR Status | | | | | |
| ER and PR positive | 900 (32%) | 224 (28%) | 175 (36%) | 378 (35%) | 123 (31%) |
| ER or PR negative | 524 (19%) | 156 (19%) | 99 (20%) | 197 (18%) | 72 (18%) |
| ER and PR negative | 1,257 (45%) | 405 (50%) | 201 (41%) | 466 (43%) | 185 (46%) |
| Missing | 96 (3%) | 19 (2%) | 15 (3%) | 39 (4%) | 23 (6%) |
| Cardiovascular Disease | | | | | |
| Absent | 2,015 (73%) | 577 (72%) | 383 (78%) | 776 (72%) | 279 (69%) |
| Present | 762 (27%) | 227 (28%) | 107 (22%) | 304 (28%) | 124 (31%) |
| Coronary Artery Disease | | | | | |
| Absent | 2,730 (98%) | 790 (98%) | 486 (99%) | 1,061 (98%) | 393 (98%) |
| Present | 47 (2%) | 14 (2%) | 4 (1%) | 19 (2%) | 10 (2%) |
| Cerebrovascular Disease | | | | | |
| Absent | 2,769 (100%) | 802 (100%) | 489 (100%) | 1,076 (100%) | 402 (100%) |
| Present | 8 (0%) | 2 (0%) | 1 (0%) | 4 (0%) | 1 (0%) |
| Heart Failure | | | | | |
| Absent | 2770 (100%) | 803 (100%) | 489 (100%) | 1,079 (100%) | 399 (99%) |
| Present | 7 (0%) | 1 (0%) | 1 (0%) | 1 (0%) | 4 (1%) |
| Arrhythmia | | | | | |
| Absent | 2,728 (98%) | 797 (99%) | 489 (100%) | 1,050 (97%) | 392 (97%) |
| Present | 49 (2%) | 7 (1%) | 1 (0%) | 30 (3%) | 11 (3%) |
| Other cardiovascular diseases | | | | | |
| Absent | 2734 (98%) | 796 (99%) | 485 (99%) | 1061 (98%) | 392 (97%) |
| Present | 43 (0%) | 8 (1%) | 5 (1%) | 19 (2%) | 11 (3%) |
| Hypertension | | | | | |
| Absent | 2098 (76%) | 596 (74%) | 391 (80%) | 814 (75%) | 297 (74%) |
| Present | 679 (24%) | 208 (26%) | 99 (20%) | 266 (25%) | 106 (26%) |
| Beta-blocker | | | | | |
| Not Taking | 2,511 (90%) | 744 (93%) | 439 (90%) | 977 (90%) | 351 (87%) |
| Taking | 266 (10%) | 60 (7%) | 51 (10%) | 103 (10%) | 52 (13%) |
| Diabetes | | | | | |
| Absent | 2,546 (92%) | 737 (92%) | 459 (94%) | 982 (91%) | 368 (91%) |
| Present | 231 (8%) | 67 (8%) | 31 (6%) | 98 (9%) | 35 (9%) |
| Prior Taxane all settings | | | | | |
| No | 1347 (49%) | 620 (77%) | 0 | 724 (67%) | 0 |
| Yes | 1430 (51%) | 184 (23%) | 488 (100%) | 356 (33%) | 402 (100%) |
| Prior Anthracyclines all settings | | | | | |
| No | 1461 (53%) | 491 (61%) | 190 (39%) | 599 (55%) | 181 (45%) |
| Yes | 1316 (47%) | 313 (39%) | 300 (61%) | 481 (45%) | 222 (55%) |
| Prior Trastuzumab all settings | | | | | |
| No | 1454 (52%) | 716 (89%) | 0 | 738 (68%) | 0 |
| Yes | 1323 (48%) | 88 (11%) | 490 (100%) | 342 (32%) | 403 (100%) |
| CI=confidence interval, HR=hazard ratio, BMI = Body Mass Index, WHO = World Health Organization, ER = Estrogen Receptor, PR = Progesterone Receptor, ECOG PS = Eastern Cooperative Oncology Group Performance Status, LLN = Lower Limit of Normal | | | | | |

Supplementary Table 2: Patient characteristics by status of BB use

| **Variable** | **Total** | **Non-users** | **Users** |
| --- | --- | --- | --- |
| Total | 2777 (100%) | 2511 (90.4%) | 266 (9.6%) |
| Age |  |  |  |
| <=60 | 2068 (74.5%) | 1932 (76.9%) | 136 (51.1%) |
| >60 | 709 (25.5%) | 579 (23.1%) | 130 (48.9%) |
| Race |  |  |  |
| Non-Asian | 2113 (76.1%) | 1878 (74.8%) | 235 (88.3%) |
| Asian | 663 (23.9%) | 632 (25.2%) | 31 (11.7%) |
| Missing | 1 (0%) | 1 (0%) | 0 (0%) |
| BMI - WHO classification |  |  |  |
| Normal | 1199 (43.7%) | 1123 (45.2%) | 76 (28.9%) |
| Obese | 627 (22.8%) | 522 (21%) | 105 (39.9%) |
| Overweight | 846 (30.8%) | 766 (30.8%) | 80 (30.4%) |
| Underweight | 74 (2.7%) | 72 (2.9%) | 2 (0.8%) |
| Missing | 31 (1.1%) | 28 (1.1%) | 3 (1.1%) |
| ECOG PS |  |  |  |
| 0 | 1703 (61.4%) | 1568 (62.5%) | 135 (50.8%) |
| >=1 | 1071 (38.6%) | 940 (37.5%) | 131 (49.2%) |
| Missing | 3 (0.1%) | 3 (0.1%) | 0 (0%) |
| ER & PR Status |  |  |  |
| ER and PR positive | 900 (33.6%) | 814 (33.5%) | 86 (33.9%) |
| ER or PR negative | 524 (19.5%) | 474 (19.5%) | 50 (19.7%) |
| ER and PR negative | 1257 (46.9%) | 1139 (46.9%) | 118 (46.5%) |
| Missing | 96 (3.5%) | 84 (3.3%) | 12 (4.5%) |
| Brain metastasis |  |  |  |
| No | 2676 (96.5%) | 2420 (96.5%) | 256 (96.2%) |
| Yes | 98 (3.5%) | 88 (3.5%) | 10 (3.8%) |
| Missing | 3 (0.1%) | 3 (0.1%) | 0 (0%) |
| Albumin |  |  |  |
| < LLN | 268 (9.8%) | 228 (9.3%) | 40 (15.3%) |
| >= LLN | 2455 (90.2%) | 2233 (90.7%) | 222 (84.7%) |
| Missing | 54 (1.9%) | 50 (2%) | 4 (1.5%) |
| Cardiovascular disease |  |  |  |
| No | 2015 (72.6%) | 1966 (78.3%) | 49 (18.4%) |
| Yes | 762 (27.4%) | 545 (21.7%) | 217 (81.6%) |
| Diabetes |  |  |  |
| No | 2546 (91.7%) | 2335 (93%) | 211 (79.3%) |
| Yes | 231 (8.3%) | 176 (7%) | 55 (20.7%) |
| Visceral Disease |  |  |  |
| Absent | 778 (28%) | 690 (27.5%) | 88 (33.1%) |
| Present | 1999 (72%) | 1821 (72.5%) | 178 (66.9%) |
| Prior Anthracycline in all settings |  |  |  |
| No | 1461 (52.6%) | 1328 (52.9%) | 133 (50%) |
| Yes | 1316 (47.4%) | 1183 (47.1%) | 133 (50%) |
| Prior Trastuzumab in all settings |  |  |  |
| No | 1454 (52.4%) | 1339 (53.3%) | 115 (43.2%) |
| Yes | 1323 (47.6%) | 1172 (46.7%) | 151 (56.8%) |
| Prior Taxane in all settings |  |  |  |
| No | 1347 (48.5%) | 1232 (49.1%) | 115 (43.2%) |
| Yes | 1430 (51.5%) | 1279 (50.9%) | 151 (56.8%) |
| BMI = Body Mass Index, WHO = World Health Organization, ER = Estrogen Receptor, PR = Progesterone Receptor, ECOG PS = Eastern Cooperative Oncology Group Performance Status, LLN = Lower Limit of Normal | | | |

Supplementary Table 3: Univariable and adjusted analysis of survival outcomes

|  |  | **Overall Survival** | | **Progression-free survival** | |
| --- | --- | --- | --- | --- | --- |
|  |  | **Univariable** | **Adjusted** | **Univariable** | **Adjusted** |
| **Variables** | **N** | **HR [95% CI]** | **HR [95% CI]** | **HR [95% CI]** | **HR [95% CI]** |
| Beta-blocker at baseline |  |  |  |  |  |
| No | 2511 | 1 | 1 | 1 | 1 |
| Yes | 266 | 1.33[1.12-1.58] | 1.26[1.03-1.54] | 1.11[0.96-1.29] | 1.08[0.91-1.28] |
| Age |  |  |  |  |  |
| <=60 | 2068 | 1 | 1 | 1 | 1 |
| >60 | 709 | 1.09[0.96-1.23] | 1.00[0.87-1.15] | 0.99[0.89-1.09] | 0.95[0.84-1.06] |
| Race |  |  |  |  |  |
| Non-Asian | 2113 | 1 | 1 | 1 | 1 |
| Asian | 663 | 0.89[0.78-1.02] | 0.79[0.68-0.91] | 0.91[0.81-1.01] | 0.84[0.75-0.95] |
| BMI - WHO classification |  |  |  |  |  |
| Normal | 1199 | 1 | 1 | 1 | 1 |
| Obese | 627 | 0.90[0.78-1.04] | 0.81[0.69-0.95] | 0.89[0.79-0.99] | 0.84[0.74-0.95] |
| Overweight | 846 | 0.91[0.80-1.04] | 0.87[0.75-0.99] | 0.93[0.84-1.03] | 0.91[0.81-1.01] |
| Underweight | 74 | 1.17[0.83-1.64] | 1.30[0.92-1.84] | 1.19[0.91-1.57] | 1.31[0.99-1.74] |
| Albumin |  |  |  |  |  |
| < LLN | 268 | 1 | 1 | 1 | 1 |
| >= LLN | 2455 | 0.53[0.45-0.63] | 0.59[0.50-0.71] | 0.67[0.58-0.77] | 0.71[0.62-0.83] |
| ER & PR Status |  |  |  |  |  |
| ER and PR positive | 900 | 1 | 1 | 1 | 1 |
| ER or PR negative | 524 | 1.06[0.90-1.25] | 1.05[0.89-1.24] | 1.02[0.89-1.16] | 1.01[0.89-1.15] |
| ER and PR negative | 1257 | 1.35[1.19-1.54] | 1.40[1.22-1.60] | 1.02[0.92-1.13] | 1.04[0.94-1.16] |
| ECOG PS |  |  |  |  |  |
| 0 | 1703 | 1 | 1 | 1 | 1 |
| >=1 | 1071 | 1.64[1.47-1.83] | 1.59[1.41-1.79] | 1.38[1.26-1.51] | 1.37[1.25-1.51] |
| Visceral Disease |  |  |  |  |  |
| Absent | 778 | 1 | 1 | 1 | 1 |
| Present | 1999 | 1.53[1.34-1.74] | 1.48[1.29-1.70] | 1.29[1.16-1.43] | 1.26[1.13-1.40] |
| Brain metastasis |  |  |  |  |  |
| No | 2676 | 1 | 1 | 1 | 1 |
| Yes | 98 | 1.66[1.29-2.14] | 1.13[0.86-1.49] | 1.32[1.05-1.66] | 1.04[0.81-1.33] |
| Diabetes |  |  |  |  |  |
| No | 2546 | 1 | 1 | 1 | 1 |
| Yes | 231 | 1.11[0.91-1.35] | 1.10[0.89-1.37] | 0.97[0.82-1.14] | 1.01[0.85-1.20] |
| Cardiovascular disease |  |  |  |  |  |
| No | 2015 | 1 | 1 | 1 | 1 |
| Yes | 762 | 1.12[1.00-1.27] | 1.03[0.88-1.19] | 0.98[0.89-1.08] | 0.97[0.86-1.10] |
| Prior Taxane in all settings |  |  |  |  |  |
| No | 1347 | 1 | 1 | 1 | 1 |
| Yes | 1430 | 1.12[0.96-1.31] | 1.01[0.82-1.25] | 1.06[0.94-1.20] | 0.94[0.79-1.10] |
| Prior Anthracycline in all settings |  |  |  |  |  |
| No | 1461 | 1 | 1 | 1 | 1 |
| Yes | 1316 | 1.21[1.08-1.35] | 1.21[1.06-1.137] | 1.07[0.98-1.17] | 1.02[0.92-1.14] |
| Prior Trastuzumab in all settings |  |  |  |  |  |
| No | 1454 | 1 | 1 | 1 | 1 |
| Yes | 1323 | 1.16[0.97-1.39] | 1.02[0.81-1.27] | 1.21[1.06-1.39] | 1.25[1.06-1.49] |
| CI=confidence interval, HR=hazard ratio, BMI = Body Mass Index, WHO = World Health Organization, ER = Estrogen Receptor, PR = Progesterone Receptor, ECOG PS = Eastern Cooperative Oncology Group Performance Status, LLN = Lower Limit of Normal | | | | | |

Supplementary Table 4: Subset analysis by STUDY to evaluate the effect of BB on survival outcomes

|  |  |  | **Overall Survival Progression Free Survival** | | | |
| --- | --- | --- | --- | --- | --- | --- |
| **Subgroup** | **Variable** | **N** | **HR [95% CI]** | **P** | **HR [95% CI]** | **P** |
| CLEOPATRA | Beta-blocker use^1^ | | | 0.636 |  | 0.927 |
|  | No | 744 | 1 |  | 1 |  |
|  | Yes | 60 | 1.11 [0.73 to 1.68] |  | 0.98 [0.70 to 1.39] |  |
| MARIANNE | Beta-blocker use^2^ | | | 0.381 |  | 0.565 |
|  | No | 977 | 1 |  | 1 |  |
|  | Yes | 103 | 1.19 [0.81 to 1.74] |  | 1.09 [0.81 to 1.46] |  |
| TH3RESA | Beta-blocker use^3^ | | | 0.255 |  | 0.853 |
|  | No | 351 | 1 |  | 1 |  |
|  | Yes | 52 | 1.58 [0.99 to 2.53] |  | 1.04 [0.68 to 1.60] |  |
| EMILIA | Beta-blocker use^3^ | | | 0.306 |  | 0.628 |
|  | No | 439 | 1 |  | 1 |  |
|  | Yes | 51 | 1.23 [0.83 to 1.84] |  | 1.10 [0.74 to 1.63] |  |
| 1. Adjustment Variables: Age, Race, BMI, Albumin count, ECOG PS, ER/PR Status, Presence of Visceral Disease, Coronary Artery Disease, Cerebrovascular disease, Arrhythmia, Hypertension, Diabetes and other Cardiovascular diseases, Prior treatment to anthracyclines, taxanes and trastuzumab. 2. Adjustment Variables: Age, Race, BMI, Albumin count, ECOG PS, ER/PR Status, Presence of Visceral Disease and Brain Metastasis, Presence of Visceral Disease, Coronary Artery Disease, Cerebrovascular disease, Arrhythmia, Hypertension, Diabetes and other cardiovascular diseases, Prior treatment to anthracyclines, taxanes and trastuzumab. 3. Adjustment Variables: Age, Race, BMI, Albumin count, ECOG PS, ER/PR Status, Presence of Visceral Disease, Coronary Artery Disease, Cerebrovascular disease, Arrhythmia, Hypertension, Presence of Visceral Disease and Brain Metastasis, Diabetes and other cardiovascular diseases, Prior treatment to anthracyclines.   CI= Confidence interval, HR= Hazard ratio, N = Number of subjects, ECOG PS= Eastern cooperative oncology group performance status, LLN= Lower limit of normal, BMI = Body Mass Index & ER/PR = Estrogen Receptor/Progesterone Receptor | | | | | | |
